# A Boycotted Hospital

**Published:** 1918-08-31

**Authors:** 


### A Boycotted Hospital.

A GRAVE state of affairs in connection with the Launceston General Hospital, Tasmania, has followed upon the quarrel between the British Medical Association and the Government, in respect of the treatment of affluent patients by the hospitals. The house surgeon of the institution having resigned, every endeavour has been made to obtain the services of a duly qualified medical practitioner to replace him, but with no success, the British Medical Association having barred any of its members from accepting the vacant appointment. The board of management have only the services of the surgeon-superintendent (Dr. Sweetman) to rely upon, and he continues to look after the 150 indoor patients. It will be remembered that the honorary staff of this hospital resigned last year. In the circumstances it was inevitable that some part of the work would have to be discontinued, should the paid staff be diminished; it is therefore not surprising to learn that the out-patient department has been closed. This is very regrettable, inasmuch as the out-patients include men, women, and children who are unable to pay either for medical treatment or medicine, which has hitherto been supplied to them by the hospital. A conference between representatives of the medical profession and the board has been agreed to, and it seems probable that some solution to the difficulty, if but temporary, will result.

